# Clinical Efficacy of *Moringa oleifera* Lam. Stems Bark in Urinary Tract Infections

**DOI:** 10.1155/2014/906843

**Published:** 2014-11-02

**Authors:** Santosh Kumar Maurya, Anil Kumar Singh

**Affiliations:** ^1^Ayurvedic Pharmacy Laboratory, Banaras Hindu University, Rajiv Gandhi South Campus, Barkachha, Mirzapur 231001, India; ^2^Department of Dravyaguna, Faculty of Ayurveda, Institute of Medical Sciences, Banaras Hindu University, Varanasi 221005, India

## Abstract

*Objective*. Urinary tract infections (UTI) are the most common problem in clinical practice. Usually they are asymptomatic and are commonly present with distressing symptoms like pain and burning sensation on urination. Antibiotics are widely used to treat UTIs; however, they have their own limitations like resistance, reinfection, and relapses. The purpose of the current study was to evaluate the value of *Moringa oleifera* Lam. stem bark as a potential medicine for UTIs. *Study Design*. 30 patients with UTI were randomly divided into two groups with 15 patients in each group. Shigru bark was given to patients of the first group (trial group) and modern medicines were prescribed to the other group of patients. At least three follow-ups are taken in both groups at the end of every week of treatment. *Results*. After treatment 66.67 % were cured, 13.33 % improved, 13.33% patients have no change, and 6.67% relapsed in trial group and in control group 46.67% were cured, 26.66% improved, 6.67% patients have no change, and 20% relapsed. *Interpretation and Conclusion*. The trial drug is significant in the management of UTI. This study needs to be done on a large scale and for a long time.

## 1. Introduction 

Urinary tract infections (UTIs) account for 8.3 million doctor visits yearly in the United States and are the second most common type of infection in the body. It is one of the most serious health problems affecting millions of people each year [1]. UTIs include acute as well as chronic cystitis, prostatitis, pyelonephritis, and urethritis. Lower urinary tract infection is more common among women with an incidence of one in five women at any point throughout their life. Men are usually devoid of UTIs, but sometimes due to an obstruction such as a stone or enlarged prostate they experience this. Prevalence of UTIs in men under 50 years is uncommon [2]. Although most of the cases of UTI are asymptomatic or with a few distressing complaints like frequency, urgency of urination, pain during urination, difficulty in passing urine, and feeling of heaviness or pain in the pubic region even at rest, different anatomic (obstructing lesions) and physiologic (estrogen deficiency, genetic factors, and regular antibiotic coverage) factors are predisposing factors for UTIs. Anal intercourse and HIV infection also contribute to infection of urinary tract [3, 4].

The majority of causative organisms of UTI are gram-negative bacteria in which* Escherichia coli* alone contribute to 80 percent of cases.* Proteus mirabilis*,* Klebsiella pneumonia, *and* Enterobacter aerogenes *are also involve in the pathogenesis of the disease. Gram-positive bacteria include* Staphylococcus saprophyticus* (10–15%),* Enterococci*, and* Staphylococcus aureus* (associated with calculi and catheterization). Microbiologically, UTI is defined as presence of at least 10^5^ organisms/mL of urine in an asymptomatic patient or as more than 100 organisms/mL of urine in a symptomatic patient with accompanying pyuria (>5 WBCs/mL) [5]. Conventional antibiotics are the first choice in an acute episode of UTI; therefore resistance of pathogenic bacteria to antibiotics is of high clinical concern. The concept of the control of drug resistance is rather widely held today [6, 7]. Several reports are available about the multidrug resistance of bacteria especially* Staphylococcus, Pseudomonas, and Escherichia.* Therefore the world looks at some alternative and effective medicine particularly of natural origin. Some of the clinical studies recommend that mannose, cranberry, and probiotics can be natural options for long-term prevention from UTIs [2]. Ayurveda and some other conventional systems of medicines state that plants are main reservoirs of natural entities which have been used for the treatment of different ailments. It was reported that over 70% of the Indian people believe in nature for their food as well as medicines. These natural medicines play a vital role in primary health care facilities [8].

The Moringaceae is a single-genus family of oil seed trees with 14 well known species.* Moringa oleifera*, commonly known as horseradish tree or drumstick tree and Saijan in Hindi, ranges in height from 5 to 10 m [9, 10]. It is indigenous to sub-Himalayan regions of northwest India, Africa, Arabia, Southeast Asia, and the Pacific and Caribbean Islands [9].* M. oleifera* is known as nutritional supplement with a variety of medicinal properties. The drumstick tree was used as food and medicine for centuries; it is only recently that scientific basis for these uses has been recognized.

The stem bark has been reported to contain two alkaloids, namely, moringine and moringinine, vanillin, *β*-sitosterol, *β*-sitostenone, 4-hydroxymellin, and octacosanoic acid [11] and phenolics [12]. Several procyanidin [13] and 4-(-l-rhamnopyranosyloxy)-benzylglucosinolate [14] also reported from the same. Its bark possesses antibacterial [15–21] activities against varieties of both gram-positive and gram-negative bacteria, antifungal [20], anticholesteremic, antilipidemic [22], antifertility [22], anti-inflammatory, diuretic [23], antiurolithiatic [24], antioxidant [12], antisickling [25], cardioprotective [26], hepatoprotective [27], and hypoglycaemic [28] effect. It is also used for treatment of fractured bones [29]. The bark was found to be safe in animal toxicity study [30]. The aim of the present study was to find out the efficacy of* M. oleifera* stem bark as a potential treatment for urinary tract infection.

## 2. Material and Methods 

### 2.1. Selection of Sample

A total of 30 patients were registered from the Dravyaguna Out Patient Department, Ayurveda Wing, Sir Sunderlal Hospital, Institute of Medical Sciences, Banaras Hindu University, Varanasi, after proper screening on the predesigned pro forma. The cases were selected, also, on the basis of the following inclusion (adult, uncomplicated UTI, and patients with BPH) and exclusion (patients with renal failure, calculus, and stricture or any structural pathology and pregnant women) criteria. Then all the cases are screened for other possible causes of UTI before including in the final sample. Screening of each patient's details was noted on preformed format. The work has been approved by the Ethical Committee of the Institute of Medical Sciences, Banaras Hindu University, Varanasi (Ref: Dean/2010-11/92 dated: 5th May 2010).

### 2.2. Collection and Preparation of Medicine

The drug was collected from its natural habitat from Varanasi. Identification of drug was carried out in the Department of Dravyaguna, Faculty of Ayurveda, Institute of Medical Sciences, Banaras Hindu University, Varanasi. The stem bark is powdered coarsely for decoction in Ayurvedic Pharmacy, Faculty of Ayurveda, Institute of Medical Sciences, Banaras Hindu University, Varanasi.

### 2.3. Clinical Study

30 patients with UTI were randomized and divided into two groups, 15 patients in each. One group is treated with trial drug Shigru bark decoction; 40 mL (made by boiling 15 g of coarse powder in 100 mL water) for 21 days, twice daily, is given to patients for treatment; another group was named control group in which standard modern medication was administered.

To assess the effect, other drugs were given randomly without selection of patients. But after getting investigations and effect of the drugs on subsequent follow-ups (responding/not responding), these cases were classified also as per the characteristic of urine, sign/symptoms concerned with the specific pathogen grown in the urine. Patients were also advised to drink lukewarm water, avoid sexual intercourse, suppression of natural urge of urination, exercise, excessive speech, uneven sitting and lying postures, exposure to wind, cold, heat, and dust, anger, and grief.

Patients of both groups were advised to come for follow-up every 7 days. At every follow-up changes in clinical features were noted and the investigations were repeated. The patient of test group was advised to continue the oral medication that was continued for the next 7 days. In control group patients were advised to continue the prescribed antibiotic in the therapeutic doses for the duration of the next 15 days. All these patients were followed for at least two months. Those cases with a minimum of the three follow-ups or who had minimum treatment for duration of one and half months were included for analysis of result.

### 2.4. Assessment of Improvement in Condition

Assessment was made on the following criteria during follow-up:

1. changes in clinical symptomatology (Table 1);
2. finding in the routine and microscopic examination of urine;
3. finding in urine culture.

### 2.5. Statistical Analysis

Descriptive statistical analysis has been carried out in the present study. Results on continuous measurements are presented on mean ± SD and results on categorical measurements are presented in number (%). Significance is assessed at 5% level of significance. Data were analyzed by chi-square test and Student's *t*-test.

## 3. Result and Discussion

### 3.1. Demographic Profile

The age distribution of the patients in the sample set was 25–65 years (mean age of 36.16). The prevalence of UTI was highest within 25–35 years (30%) followed by 35–45 years (20%), 15–25 years (20%), 45–55 years (16.67%), and 55–65 years (13.33%). The majority of cases were female (56.67%) and 43.33% of cases were male patients which is in agreement with earlier studies [31–35]. The peculiarities of female anatomy with shorter urethra and closeness of the urethral meatus and anus have been reported as factors which influence the higher prevalence in women [36]. The age group analysis showed that young patients usually female in the range of 25–35 years had high prevalence rate of UTI. This result is in agreement with previous studies [37, 38] which shows that among sexually active young women the incidence of symptomatic UTI is high and the threat is firmly connected with sexual intercourse, fresh utilization of diaphragm with spermicide, and a positive history of recurrent UTIs. The majority of cases were married (70%), followed by 7 (23.33%) unmarried and 2 (6.67%) widows. The majority of cases were Hindu (90%); only 10% of cases were Muslim as circumcision seems to reduce the overall incidence of UTI in Muslims [39]. Prevalence of disease is highest in business and housewife (both 26.67%) then followed by student (16.67%) farmers (13.33%), service class (10%) and unemployed (6.66%) [40, 41]. The majority of cases belonged to rural area (53.33%) followed by urban area (46.67%). A maximum number of cases are from the disease that were of middle class (73.33%) followed by lower (20%) and upper (6.67%) [42].

It was observed that total duration of illness ranged from 6 month-1 year (33.33%) to 5 years (13.33%). Clinical profile of these patients exhibited fever (80%), oliguria (70%), burning urination (66.67%), frequency (63.33%), pain, and urgency (60%). 46.67% patients with hesitancy and tenderness followed by constipation (20%). Routine and microscopic urine examination of these patients at starting of treatment exhibited sugar (53.33%), albumin (50%), RBC (33.33%), epithelium (26.67%), pus cell (30%) 10% patients with crystals. In our study gram-negative aerobic rods predominated, among which* E. coli* was the commonest uropathogen responsible for UTI. It accounted for 60.0% of all positive cultures, while* K. pneumoniae* and* Proteus mirabilis* were the next most common organisms, accounting for 16.67% and 10% of culture in the present study, respectively.* Pseudomonades aeruginosa, Streptococcus faecalis,* and* Staphylococcus aureus* are also present in some of the patients (Table 2). The percentage of causative bacterial class was analogous to some other previous studies [43–47].

### 3.2. Therapeutic Efficacy

In our study we found that fever was absent in only 6.67% of cases at the end of treatment in test group; however a gradual decrease in the number of patients has been observed (Table 3).  Table 4 reveals therapeutic effect within both groups in successive follow-ups, which is highly significant in group A (*P* < 0.001) and significant in group B (*P* < 0.005) after treatment (AT). Initial mean reduced from 2.88 ± 0.719 to 2.31 ± 0.704 after treatment regimen in group A and from 3.25 ± 0.577 to 2.75 ± 0.683 in group B. Intergroup comparison between before treatment (BT) and after treatment (AT) was not found to be significant. It means that the tested drug is effective in controlling fever but it is not as effective as antibiotics.

Successful reduction in burning sensation during urination was observed (Table 3) in both groups. Only in 26.67% of cases burning urination was relieved at the end of treatment in group A, while 6.67% of cases were relieved from the complaint AT in group B.  Table 4 showed effect on burning urination in both groups on successive follow-ups which is highly significant in group A (*P* < 0.001) and significant in group B (*P* < 0.005). Intergroup comparison was observed to be statistically significant in BT and AT. It means that the tested drug is effective in controlling the burning sensation during urination but not as effective as antibiotics.

Relief from pain during urination is observed in both groups. In 40% of cases pain was completely relieved at the end of treatment in group A. In group B, 6.67% of cases were relieved from the complaint AT (Table 3).  Table 4 showed significant changes in relieving pain in both groups in successive follow-ups (*P* < 0.01).

In 6.67% of cases, frequency of urination was normalized at the end of treatment in group A. In group B, 6.67% of cases were relieved from the complaint AT (Table 3).  Table 4 showed significant changes in frequency in both groups in successive follow-ups (*P* < 0.05).

Improvement in amount of urine is also observed in both groups. In 6.67% of cases, oliguria was relieved at the end of treatment in group A. In group B, 6.67% of cases were relieved from the complaint AT (Table 3).  Table 4 showed significant improvement in oliguria disorder in both groups in successive follow-ups (*P* < 0.05).

The routine and microscopic urine examination of these patients after treatment showed significant effect of the test drug. Pus cell and crystals were absent in both groups. However in some cases sugar, albumin, and epithelium were present. The culture reports showed that the herb has prominent effect on* E. coli* and other bacteria like* K. pneumoniae* and* P. mirabilis.* However,* P. aeruginosa, S. faecalis,* and* S. aureus* were not found to be present in any of the patients at the end of the treatment.

As evident from Table 5, the majority of cases were cured (56.67%) after three weeks of treatment, followed by improving (20%) and relapsing (13.33%).

This study was conducted on volunteer patients in the Department of Dravyaguna, Faculty of Ayurveda, Institute of Medical Sciences, Banaras Hindu University, Varanasi, India, spread over a period of one and half years. The study was carried out according to the institution guidelines for biomedical research involving human subjects. Our data show that decoction of* M. oleifera* is an effective remedy for symptomatic relief in UTIs. This is an encouraging result which clearly favors promotion of* M. oleifera* as a remedy among rural communities especially belonging to low socioeconomic strata as* M. oleifera* is easily accessible and is cheap and safe. The results demonstrated a significant reduction of symptoms in both groups A and B over a period of two months. Levofloxacin has been utilized for several years and it is still considered one of the most effective agents in UTIs. However, recurrence is quite common; therefore new drugs from herbal origin with similar or superior efficacy and possibly fewer long-term effects need to be investigated.* M. oleifera* is one of the common Indian herbs used for different conditions related to urinary system such as urolithiasis and benign prostrate hyperplasia. This plant has been used in India and other parts of Asia for thousands of years for nutritional as well as medical purposes. In traditional literature, preparations of the* M. oleifera* are claimed to be effective in a wide spectrum of inflammatory and infectious diseases. Stem bark of* M. oleifera* has been tested against a variety of microorganisms like* E. coli, S. aureus, B. cereus, P. aeruginosa, and P. mirabilis.* It shows prominent effect against* E. coli* (MIC 64 *μ*g/mL) [17], which is a major causative factor in the UTIs.

In the present study,* M. oleifera* decoction provides symptomatic relief to the patients during the trial; possible explanation for this effect might be due to the antibacterial and anti-inflammatory agents present in the plant [15, 17–21, 23]. Urinary stone is also one of the conditions which facilitate UTIs; therefore antiurolithiatic activity [24] of drumstick might also contribute in its action on urinary system. Urinary tract infection in addition aggravates the oxidative stress in some cases like diabetes [48] and pregnancy [49]. So antioxidant activity [12] of* M. oleifera* bark is effective against oxidative stress produced in UTI probably because of phenolic component [50].

Painful and inconvenient, a UTI results in frequent urges to urinate with little production of urine and is accompanied by a severe burning sensation. We observed increase in urine amount indicating diuretic action of Moringa [51]. Diuretic abilities of Moringa also appear to inhibit adheration of infection-causing bacteria to the wall of the bladder, so they are sloughed off in urine as reported in the similar case of cranberry [52].

However, as this is the first attempt to assess the effect of* M. oleifera* in UTIs, clinical trial of longer duration with a larger sample size should play a vital role in commercialization of* M. oleifera*.

## 4. Conclusion

From the clinical study, it has been concluded that stem bark of* M. oleifera,* at a dose of 40 mL BD, is effective on most of the cardinal symptoms of urinary tract infection. However, further investigations are required to elucidate their exact mechanism of action. The drug was well tolerated and does not generate any adverse effect during the entire clinical study. The drug is also helpful in eradicating urinary pathogens like* E. coli* that are responsible for UTI.

## Figures and Tables

**Table 1:** Score for symptom assessment.

Score	Pain during urination	Burning sensation	Urine amount (mL/24 h)	Fever	Frequency of urination
1	No pain	No burning	>1500	No fever	Normal
2	Occasional bearable pain	Occasional mild burning	1000–1500	Mild fever (99–100°F)	5–8 times
3	Often/moderate pain	Moderate troublesome burning	500–1000	Moderate fever (100–102°F)	8–12 times
4	Severe pain	Severe burning	<500	High fever (>102°F)	>12 times

**Table 2:** Showing result of urine examination in both groups before and after treatment.

	Before treatment (BT)		After treatment (AT)	
	Group A (%)	Group B (%)	Group A (%)	Group B (%)
Routine and microscopic examination				
Albumin	53.33	46.67	6.67	13.33
Sugar	66.67	40	13.33	6.67
RBC	46.67	20	6.67	00
Epithelium	26.67	26.67	6.67	6.67
Pus cell	26.67	33.33	00	00
Crystals	13.33	6.67	00	00
Culture test				
*E*. *coli *	60	60	13.33	6.67
*K*. *pneumoniae *	20	13.33	6.67	00
*P*. *mirabilis *	6.67	13.33	00	6.67
*P*. *aeruginosa *	6.67	6.67	6.67	00
*S*. *faecalis *	6.66	00	00	00
*S*. *aureus *	00	6.67	00	00

**Table 3:** Showing distribution of different symptom scores in both groups before and after treatment.

	Score	Fever		Burning micturition		Pain		Frequency		Oliguria	
		BT	AT	BT	AT	BT	AT	BT	AT	BT	AT
Group A	1 2 3 4	00 33.33 46.67 20	6.67 60 26.67 6.67	00 20 46.67 33.33	26.67 46.67 26.67 00	6.67 40 40 20	40 53.33 6.67 00	00 40 46.67 13.33	6.67 46.67 40 6.67	00 26.67 46.67 26.67	6.67 26.67 53.33 13.33

Group B	1 2 3 4	00 6.67 60 33.33	00 33.33 53.33 13.33	00 46.67 40 13.33	6.67 60 20 13.33	00 40 53.33 6.67	6.67 60 33.33 00	00 33.33 46.67 20	6.67 40 46.67 6.67	00 33.33 46.67 20	6.67 40 53.33 00

**Table 4:** Showing therapeutic effect on different symptoms in both groups before and after treatment.

	Fever		Burning micturition		Pain		Frequency		Oliguria	
	Group A	Group B	Group A	Group B	Group A	Group B	Group A	Group B	Group A	Group B
BT	2.88 ± 0.719	3.25 ± 0.577	3.13 ± 0.743	2.67 ± 0.724	2.80 ± 0.775	2.67 ± 0.617	2.73 ± 0.704	2.87 ± 0.743	3.00 ± 0.756	2.87 ± 0.743
AT	2.31 ± 0.704	2.75 ± 0.683	2.00 ± 0.755	2.40 ± 0.213	1.66 ± 0.617	2.26 ± 0.593	2.46 ± 0.743	2.53 ± 0.743	2.733 ± 0.798	2.466 ± 0.639
Within the group Paired *t*-test value BT-AT	3.567, *P* < 0.01	2.739, *P* < 0.05	5.90, *P* < 0.01	2.256, *P* < 0.05	6.859, *P* < 0.01	3.055, *P* < 0.01	2.256, *P* < 0.05	2.646, *P* < 0.05	2.256, *P* < 0.05	2.449, *P* < 0.05
Mean difference	0.562 ± 0.629	0.500 ± 0.730	1.133 ± 0.743	0.267 ± 0.457	1.133 ± 0.639	0.400 ± 0.507	0.266 ± 0.457	0.333 ± 0.487	0.266 ± 0.457	0.400 ± 0.632
Between the group comparison independent sample *t*-test on difference of BT and AT	0.263 *P* > 0.794		3.845 *P* < 0.01		3.479 *P* > 0.05		0.386 *P* > 0.05		0.661 *P* > 0.05	

**Table 5:** Showing result in total cases and in both groups.

Result	Total cases (%)	Group A (%)	Group B (%)	*χ* ^2^ test
Cured	56.67	66.67	46.67	2.529 *P* > 0.05
Improved	20	13.33	26.67	
No change	10	13.33	6.67	
Relapsed	13.33	6.67	20	
